# Individual Response to Botulinum Toxin Therapy in Movement Disorders: A Time Series Analysis Approach

**DOI:** 10.3390/toxins14080508

**Published:** 2022-07-24

**Authors:** Bernd Leplow, Johannes Pohl, Julia Wöllner, David Weise

**Affiliations:** 1 Department of Psychology, Martin Luther University Halle-Wittenberg, D-06120 Halle, Germany; johannes.pohl@psych.uni-halle.de; 2 MEU—Study Center of DIPLOMA University of Applied Science, D-39114 Magdeburg, Germany; woellner.julia@yahoo.de; 3 Department of Neurology, Asklepios Fachklinikum Stadtroda, Bahnhofstr. 1a, D-07646 Stadtroda, Germany; da.weise@asklepios.com; 4 Department of Neurology, University of Leipzig, Liebigstraße 23, D-04105 Leipzig, Germany

**Keywords:** dystonia, hemifacial spasm, botulinum neurotoxin, ARIMA single-case autoregressive integrated moving average, adherence

## Abstract

On a group level, satisfaction with botulinum neurotoxin (BoNT) treatment in neurological indications is high. However, it is well known that a relevant amount of patients may not respond as expected. The aim of this study is to evaluate the BoNT treatment outcome on an individual level using a statistical single-case analysis as an adjunct to traditional group statistics. The course of the daily perceived severity of symptoms across a BoNT cycle was analyzed in 20 cervical dystonia (CD) and 15 hemifacial spasm (HFS) patients. A parametric single-case autoregressive integrated moving average (ARIMA) time series analysis was used to detect individual responsiveness to BoNT treatment. Overall, both CD and HFS patients significantly responded to BoNT treatment with a gradual worsening of symptom intensities towards BoNT reinjection. However, only 8/20 CD patients (40%) and 5/15 HFS patients (33.3%) displayed the expected U-shaped curve of BoNT efficacy across a single treatment cycle. CD (but not HFS) patients who followed the expected outcome course had longer BoNT injection intervals, showed a better match to objective symptom assessments, and were characterized by a stronger certainty to control their somatic symptoms (i.e., internal medical locus of control). In addition to standard evaluation procedures, patients should be identified who do not follow the mean course-of-treatment effect. Thus, the ARIMA single-case time series analysis seems to be an appropriate addition to clinical treatment studies in order to detect individual courses of subjective symptom intensities.

## 1. Introduction

Botulinum neurotoxin (BoNT) treatment is the first-line therapy of focal dystonias such as cervical dystonia (CD) but is also used in other conditions such as hemifacial spasm (HFS), cerebral palsy and other neurogenic joint contractures. It typically leads to a U-shaped curve across injection intervals with a maximum effect at about four weeks after injection, followed by a gradual loss of effectiveness over appr. 12 weeks. This robust effect is based on mean values which ignore individual variations in efficacy. As a result, the positive effect on objective motor function is, in many patients, not reflected by subjective symptom severity ratings [1]. Many studies show that these ratings are more strongly associated with psychological variables than with patients’ motor function [2,3,4,5,6,7].

Thus, the strong impact of emotional factors, including pain control on movement disorder symptoms, is undisputed. Consequently, individual reactions to BoNT treatment cannot be adequately explained by pharmacological factors alone. Despite that, the duration of BoNT treatment is usually about 12 weeks without having an objective algorithm to lengthen or to shorten the interval between injection time points due to the patient’s personal needs. Contemporary personalized medicine, however, increasingly focuses on individual preconditions and personal reactions to standardized treatments, which have been proven successful for the majority of the respective patient groups.

Standard statistical procedures are usually based on mean values and their standard errors. Additionally, although most CD and HFS patients improve under BoNT, a considerable number that do not display the expected outcome are camouflaged within group statistics. Therefore, we used a single-case evaluation, using a parametric time series analysis in each case, based on daily symptom severity ratings to investigate whether the mean-based outcome statistics can be supplemented by a method of statistical analysis that is founded on the course of symptom development in each individual patient.

## 2. Methods

The study was approved by the Ethics Committee of the University Hospital Leipzig (reference no.: 014/18-ek). All patients gave informed consent according to the Declarations of Helsinki. We recruited 20 CD patients and 15 HFS patients from the outpatient clinic at the Department of Neurology of the University Hospital Leipzig and analyzed the individual course of subjective symptom intensity ratings between two injections with BoNT.

**Figure 1:**
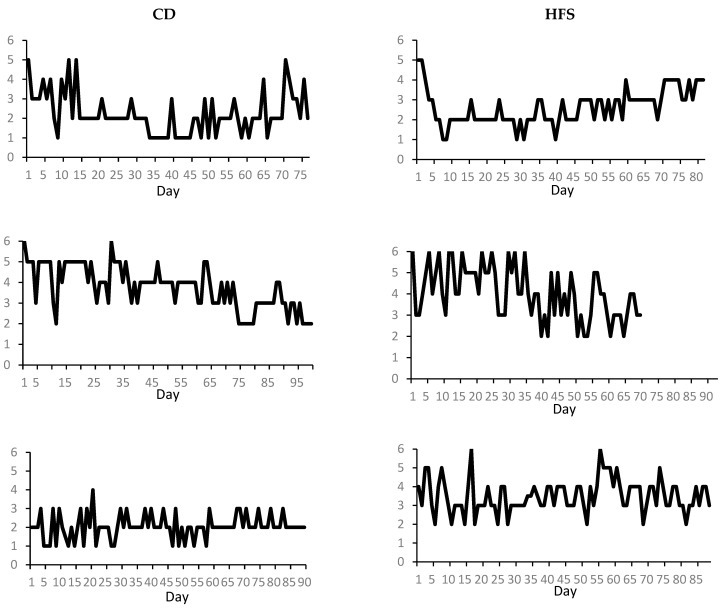
Sample graphs of daily perceived symptom intensity scores in selected CD patients (**left column**) and HFS patients (**right column**) with expected U-shaped courses (first row, d = 2), continuous worsening (second row, d = 1), and no detectable trends (third row, d = 0).

Each patient was assessed at four time points during one BoNT cycle (first injection (t1), four weeks later when it was deemed maximum BoNT efficacy was reached (t2), two weeks before the subsequent injection (t3), and at the time of BoNT reinjection (t4). At t1, patients were assessed by applying the Tsui Score, the Toronto Western Spasmodic Torticollis Rating Scale (TWSTRS), and Craniocervical Dystonia Questionnaire (CDQ-24) for understanding their mood and health-related quality of life in CD patients [8]. For the HFS patients the Jankovic Rating Scale (HFS Score) and an adaption of the CDQ-24 was used [1,8]. Patients then kept a diary, developed by our workgroup, to record subjective symptom severity. As previously described, “severity of symptoms” is the means of perceived overall symptom intensity and perceived deviation [1]. The rating scale used the German school grade scaling system (1 = best, 6 = worst; [6]). We used a single-case time series design in which each patients received BoNT treatment. Thus, each individual case was studied within a complete BoNT cycle, i.e., immediately after BoNT injection, twice within the time course, and at the end of the BoNT cycle. Analysis was based on subjective daily assessments of at least 63 consecutive and equidistant observations (max. = 100, median = 84). Since we could recruit neither CD patients nor HFS patients not receiving BoNT treatment, our single-case design was observational, with a treatment effect to be observed within each single patient. This approach is quite often used when at least 50 data points are available [9,10]. Thus, single-case autoregressive integrated moving average (ARIMA) time series analysis can be used to (i) identify the relationship between variables over time, (ii) evaluate treatment effects independent from trends or random variables, (iii) make trend predictions about forthcoming trends, and (iv) prove predicted trends on an individual basis. Here, we used the ARIMA time series analysis to test the occurrence of the expected U-shaped symptom severity function of symptom development across a single BoNT treatment in a series of single cases. The diaries were then analyzed by individual ARIMA model analysis (Figure 1), following Tabachnick’s and Fidell’s [11] recommendations. Inspecting every individual course curve, we set a first trend term parameter d. Then, autoregressive terms (p), trend terms (d), and moving average terms (q) were adapted until three criteria were met. First, autocorrelations up to lag 10 of the residual scores were negligible and the Box–Ljung test became insignificant (i.e., autocorrelation scores were not significantly different from zero). “Negligible” was defined according to Cohen’s medium effect size criterion for correlations and included scores of |r < 0.30|. Second, Pankratz’s criterion [12] |t = r_t_/SE_r_|, (rt = autocorrelation coefficient, SE_r_ = standard error of autocorrelation) was less than 1.25 for the first three lags. For lags 4 to 10, the criterion was less than 1.60 [12]. Third, partial autocorrelations of the residual scores were negligible, i.e., r < |0.30|. In the case of missing data, these scores were replaced by means from values preceding and following the missing score. This type of statistical time series analysis can adequately reflect courses and trends over time, as it has been exemplarily shown in research about mood fluctuations in bipolar disorders [13].

The ARIMA analysis was performed for each patient separately. After analysis, each patient group was divided into two sub-groups: “Responders” were defined by showing the expected U-shaped course between the injection points (d = 2 parameter, Table 1) or continuous improvement (d = 1*, parameter, Table 1). “Non-responders”, in contrast, exhibited no clear trend or a course that was continuously worsening (see Table 1, d = 0, d = 1).

Dependent variables were medical variables, sociodemographic variables, and the “medical locus of control” (mLoC; KKB, [14]). The LOC can be either internal or external. The internal mLOC refers to a belief that a person can self-influence one’s health and disease progress, whereas an external mLOC is considered when the medical disorder is thought to primarily depend on medical and social resources beyond the individual’s control; thus, individuals have little or no control or influence over the progress.

Sociodemographic variables and dystonia rating scores between the time points within BoNT treatment cycles were compared using Friedman’s test. Further inferential statistical analyses were run using Mann–Whitney U-tests and Chi² tests. Effects were considered significant if *p* < 0.05 (two-tailed).

Moreover, overestimators and underestimators were identified for each patient group. For this purpose, the respective objective and subjective assessments of disease severity at t2 was converted to z-values [1]. The differences between subjective and objective assessments were then calculated for each point of measurement. If the difference was greater than or equal to one standard deviation (SD), it was defined as “considerable”. Overestimators were defined as a subjective assessment of at least one SD higher than the objective assessment by the CD and HFS rating scale outcomes. Accordingly, for underestimators, the subjective assessment had to be at least one SD lower than the objective assessment.

## 3. Results

### 3.1. Clinical Characteristics

CD and HFS patients differed in age, but not with respect to disease duration, injection intervals, duration of BoNT therapy, satisfaction with BoNT treatment, HADS depression, or HADS anxiety scores (Table 2).

### 3.2. Mean-Based Analysis

In CD patients, perceived symptom severity ratings improved during a BoNT cycle (Chi² = 15.66, df = 3, *p* = 0.001). In contrast, HFS patients only exhibited a trend towards improvement (Chi² = 6.45, df = 3, *p* = 0.09). Regarding objective assessment, TWSTRS and Tsui scores showed a positive clinical effect in CD patients (Chi² = 28.40, df = 3, *p* < 0.0001; Chi² = 22.85, df = 3, *p* < 0.0001) and a positive HFS severity score in HFS patients, respectively (Chi² = 18.03, df = 3, *p* < 0.0001).

### 3.3. Single-Case Analysis

ARIMA single-case analysis using perceived symptom severity ratings on a daily basis showed that only 40% of CD and 33.3% of HFS patients displayed the expected U-shaped curve (Table 1, “d = 2”). Furthermore, 10/20 CD patients and 8/15 HFS patients did not display a detectable trend (d = 0) or worsened across injection points (d = 1). Two patients in each patient group showed continuously improving ratings of symptom intensities (d = 1*). Grouping these “d = 1*” patients with those showing the expected U-shaped curve, 50% of CD patients and 46.6% of HFS patients were responders, subjectively benefitting from BoNT therapy.

### 3.4. Daily Symptom Severity Ratings: Responders vs. Non-Responders

**Figure 2:**
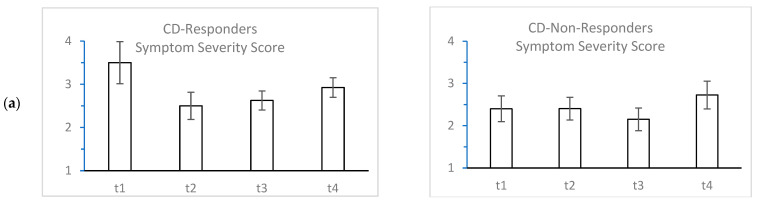

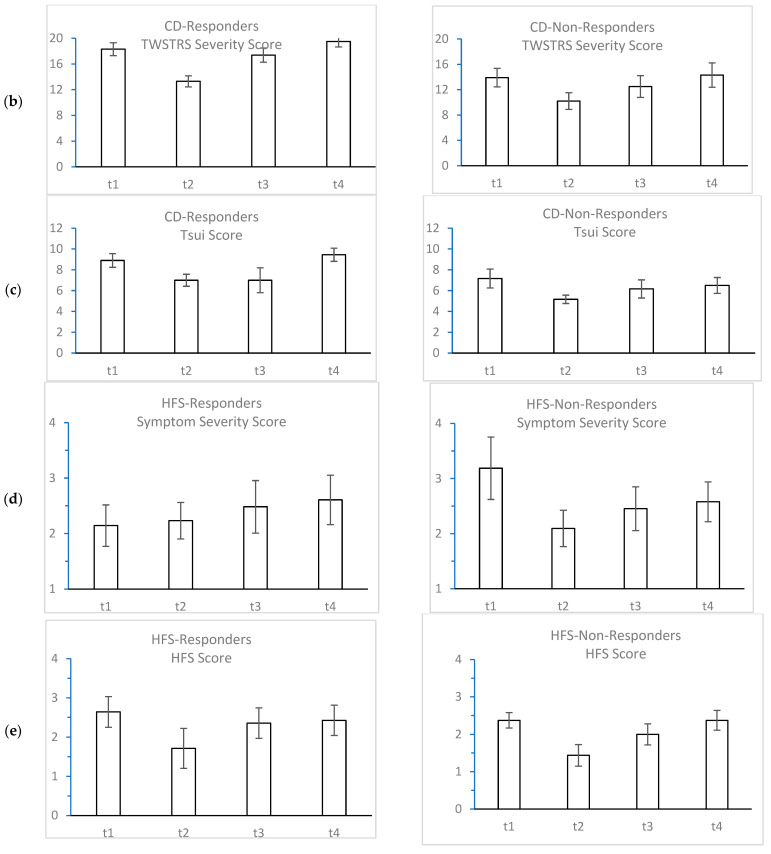
Mean scores (±standard errors) of perceived symptom intensities for responders and non-responders in CD patients (**a**–**c**) and HFS patients (**d**,**e**) at four time points across a BoNT cycle. TWSTRS Toronto Western Spasmodic Torticollis Rating Scale. HFS Score based on the Jankovic Rating Scale.

The mean ratings across four subjective and objective time points are shown in Figure 2. Objective clinical scores showed the expected U-shaped curve, irrespective of whether patients were responders or non-responders (defined by the single-case ARIMA analysis; Figure 2b,c,e, Table 3). The typical course could also be found for the subjective severity score for CD responders and HFS non-responders (Figure 2a(left),d(right), Table 3), whereas CD non-responders (Figure 2a(right)) and HFS responders did not show significant differences (Figure 2d(left)).

Responders and non-responders did not differ in age, sex, duration of disease, duration of BoNT treatment, marital status, employment status, depression and anxiety, or mental disorders prior to CD or HFS onset. CD responders showed injection intervals that were 1.2 weeks longer (U = 20.0, *p* = 0.046, Cohen’s d = 1.05, large effect size) and displayed higher internal mLoC scores (U = 1.5, *p* < 0.0001, Cohen’s d = 2.27, large effect size) compared to CD non-responders. For HFS patients, no differences between responders and non-responders were found.

### 3.5. Overestimation vs. Underestimation

In CD, all responders displayed adequate symptom perception if compared to objective ratings. Only 4 of the 10 CD non-responders showed adequate symptom perception, whereas the larger portion showed either overestimation (5/10 patients) or underestimation (1/10 patients) of their subjective symptom intensity. We detected no differences between HFS responders and non-responders regarding accordance of subjective and objective symptom assessment.

## 4. Discussion

BoNT is effective in both CD and HFS. However, our single-case analysis utilizing ARIMA model analysis revealed that only about half of participants in both patient groups actually benefitted from BoNT treatment. CD patients who responded to treatment by exhibiting the expected or continuously improving course of response to BoNT therapy displayed a better match between objective symptom assessments and subjective symptom intensity ratings, showed higher scores on the internal medical locus of control, and had a longer injection interval than CD non-responders.

Time series results were obtained by means of parametric ARIMA analysis. This method is well proven in behavioral research with experimental and quasi-experimental single-case designs [9,10,11,12,13]. Nevertheless, this method requires several preconditions. First, a parametric time series needs 30 to 50 points of measurement. Second, these time points have to be equidistant. Third, only very few missing data points are allowed, and fourth, the patients have to comply with daily assessments of comparable quality. All these conditions were fulfilled in our investigation. As seen in our study population, non-responders cannot be detected by mean score analysis (Figure 2). In addition, mean score-based interpretation alone may be misleading, because ARIMA-based dichotomization of responders and non-responders showed that even non-responders frequently display the expected U-shaped curve when only four assessment points are considered.

CD and HFS severity can vary according to emotional state [15,16,17]. It is reasonable to assume that a patient’s emotional situation immediately following the doctor’s consultation differs from their emotional state during their daily assessments over time. Therefore, assessment of the patient’s daily perception of symptom severity may give a more realistic impression of the subjective symptom development, instead of single assessments during a clinical visit.

Moreover, in small sample sizes, group means may be biased by a few outliers, but even in large sample sizes variation within a sample is of clinical significance. During statistical group testing this variation is usually reflected by measures of standard deviation or standard error. Combined with the sample mean, these measures roughly indicate treatment efficacy while neglecting clinically relevant individuals who are positioned within the upper or lower quartile of the distribution. That means that mean scores only indicate a tendency of a sample position within their respective reference group. This may explain why in our cohorts some of the non-responders mean scores also resembled the U-shaped BoNT course.

The discrepancy between the four assessments points and daily assessments may be seen within the extraordinary impact of emotional factors, which drive symptoms in movement disorders. Moreover, pain is often associated with movement disorder symptomatology. Since—besides other mediating factors—pain perception is elaborated by inhibitory stimuli and their interaction with descending pathways, as well as with their projections to the cortex, personal factors, pain perception, and the ability to behaviourally control pain responses vary widely among patients. Thus, a large number of studies has shown that in many CD patients, satisfaction with BoNT treatment and its related quality of life is widely driven by psychological mechanisms instead of objective motor improvement [2,3,4,5,6,7]. Shame, anxiety, and depression, as well as emotionally driven symptom exacerbation, are especially relevant for CD patients [15,18,19,20,21,22,23,24,25]. Both CD and HFS share the stigmatizing nature of their respective symptoms. Therefore, a daily assessment of symptom severity also reflects emotional conditions such as anxiety, depression, and hopelessness. Pain, muscular tension, and physical disability may be more closely related to pathophysiological processes of the respective disorders, whereas daily assessments over time especially reflect variations in quality of life that are only sparsely related to the physical aspects of these disorders (see [1]). Desynchronization of sensorimotor integration within the basal ganglia and their related cortical projection fields play a pivotal role both for movement control and for emotional experiences. These conditions hinder not only many activities of daily living, but are also stigmatizing. This may be the reason for the lack of subjective clinical improvement in non-responders, whereas objective assessments indicated beneficial effects of BoNT treatment. This corresponds to less internal medical locus of control, because these patients tend to follow external authorities—e.g., the doctor’s ratings at the clinical assessment. Correspondingly, these patients lack the capacity for adequate symptom perception if daily assessments are required.

Therefore, time series analysis-based trend analysis may help identify these patients. If patients’ emotional conditions are ignored, compliance and therapy adherence might decrease, and patients might choose additional complementary and alternative medical therapies (CAM) despite objective motor improvement [26,27,28]. Hence, non-motor symptoms such as depression, anxiety, and pain should be regularly assessed, e.g., using the Dystonia Non-Motor Symptoms Questionnaire [29]. Furthermore, the use of CAM therapies should be inquired about as a potential indirect parameter of treatment satisfaction and higher internal locus of control. However, not only in dystonia, but also in other conditions treated with BoNT where pain plays an important role or may even be the key symptom such as in spasticity or orthopedic conditions [30,31,32], psychological factors should be taken into account.

Assessment of individual BoNT responses over time may be beneficial for treatment planning [33,34,35,36]. Thus, longer BoNT treatment intervals or dose reduction may be appropriate in patients whose time series analysis indicates continuous symptom amelioration (d = 1*, Table 2), and ultimately individualize and optimize BoNT therapy while reducing risks. In patients with continuous worsening (d = 1, Table 2) or no clear treatment response (d = 0, Table 2), a higher dose of BoNT or shorter treatment intervals may be necessary [37].

A limitation of the study is the small sample size. Of note, a single-case analysis relies on statistical procedures within an individual person and a large number of observation points rather than on a large sample size. For a more detailed analysis, further studies should indeed cover the period of at least two treatment cycles and include groups without BoNT treatment because a single-case analysis in those patients would give insight into the dynamics of health- (and disease-)related satisfaction over time (independent from BoNT treatment). Another methodological limitation is that we only assessed subjective symptom severity for single-case analysis. This is especially relevant as we have shown in a previous study [1] that subjective and objective symptom severity ratings do not correlate well. However, an objective rating with at least 50 observation points would not have been feasible.

In summary, we propose individualized evaluation of BoNT performance as an adjunct to standard statistical group analysis. In clinical work, ARIMA time series analysis may help with treatment planning, whereas in research it seems to be an important tool to evaluate treatment effects on an individual level. In addition to mean-based analysis, parametric single-case analyses provide the investigator with information about the number of patients who do or do not benefit from a respective treatment. Adjustment of BoNT therapy may be one consequence, but especially in these patients, psychological factors should be assessed and, if possible, treated.

## Figures and Tables

**Table 1:** Number of trend characteristics of subjective symptom severity ratings following ARIMA time series analysis.

		CD	HFS
		n (%)	n (%)
d = 0	no trend detectable	9 (45.0)	6 (40.0)
d = 1	continously worsening	1 (5.0)	2 (13.3)
d = 1*	continously improving	2 (10.0)	2 (13.3)
d = 2	expected U-shaped trend	8 (40.0	5 (33.3)
Sum		20 (100.0)	15 (100.0)

Notes: “continuous worsening” and “continuous improving” are reflected by the trend (“d”) Parameter 1. To distinguished both trend types “continuous improving” was marked as d = 1*.

**Table 2:** Demographic and clinical data of CD and HFS patients.

	CD	HFD	Test Statistic
Age (years)	57.5 (14.7)	69.9 (13.5)	U = 78.0; *p* = 0.02
Sex f/m (n)	18/2	13/2	Chi² = (1) = 0.09; *p* = 0.76
Disease duration (years	10.9 (9.2)	7.8 (4.9)	U = 107.5; *p* = 0.63
Injection intervals (weeks)	11.1 (1.2)	11.0 (1.8)	U = 115.5; *p* = 0.95
Duration of BoNT therapy (years)	8.4 (8.2)	7.9 (6.2)	U = 134.5; *p* = 0.60
HADS depression	6.2 (3.8)	5.9 (3.8)	U = 113.5; *p* = 0.31
HADS anxiety	6.7 (4.4)	5.9 (3.6)	U = 131.0; *p* = 0.69
Satisfaction with BoNT therapy	2.6 (1.2)	1.9 (0.5)	U = 97.0; *p* = 0.09
Abobotulinumtoxin (n, %)	14 (70)	14 (93)	-
Incobotulinumtoxin (n, %)	6 (30)	0	-
Onabotulinumtoxin (n, %)	0	1 (7)	-

**Table 3:** Significance tests across four time points in relation to Figure 2.

	Responders	Non-Responders
CD Symptom Severity Score (Figure 2a)	10.93 (3), *p* = 0.012	6.14 (3), *p* = 0.105
HFS Symptom Severity Score (Figure 2d)	2.03 (3), *p* = 0.566	7.99 (3), *p* = 0.046
CD Tsui Score (Figure 2c)	14.57 (3), *p* = 0.002	9.75 (3), *p* = 0.021
CD TWSTRS (Figure 2b)	18.04 (3), *p* < 0.0001	10.01 (3), *p* = 0.018
HFS Score (Figure 2e)	10.27 (3), *p* = 0.016	8.84 (3), *p* = 0.032

**Notes**: Friedman tests, Chi² values, degrees of freedom, and *p*-values are given.

## Data Availability

The data presented in this study are available on request from the corresponding author.
